# Triphen­yl[(4-phenyl­benzo­yl)meth­yl]phospho­nium trifluoro­methane­sulfonate

**DOI:** 10.1107/S1600536810038286

**Published:** 2010-09-30

**Authors:** Corrado Rizzoli, Kazem Karami, Mina Mohamadi Salah

**Affiliations:** a Dipartimento di Chimica Generale ed Inorganica, Chimica Analitica, Chimica Fisica, Universitá degli Studi di Parma, Viale G. P. Usberti 17/A, I-43100 Parma, Italy; b Department of Chemistry, Isfahan University of Technology, Isfahan 84156/83111, Iran

## Abstract

In the cation of the title compound, C_32_H_26_OP^+^·CF_3_O_3_S^−^, the dihedral angle between the benzene rings of the biphenyl group is 42.37 (8)°. In the crystal, the cations and anions inter­act through inter­molecular C—H⋯O hydrogen bonds, forming chains parallel to the *b* axis. These chains are further linked by C—H⋯π stacking inter­actions into layers parallel to the *bc* plane.

## Related literature

For the synthesis and characterization of phospho­rus ylide metal complexes, see: Kalyanasundari *et al.* (1995 ▶, 1999 ▶); Laavanya *et al.* (2001 ▶); Vicente *et al.* (1985 ▶); Karami (2007 ▶, 2008 ▶); Akkurt *et al.* (2008 ▶). For related structures, see: Karami & Büyükgüngör (2009 ▶); Shao *et al.* (1982 ▶). For the synthesis of the title compound, see: Burmeister *et al.* (1973 ▶).
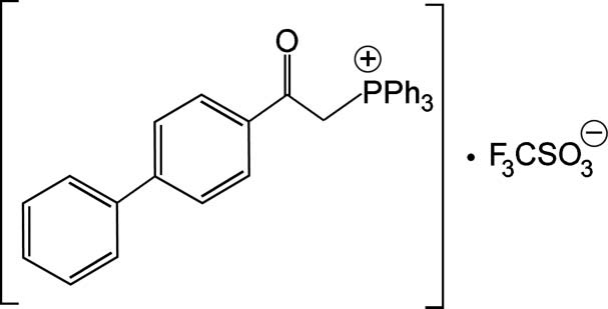


## Experimental

### 

#### Crystal data

C_32_H_26_OP^+^·CF_3_O_3_S^−^
*M* *_r_* = 606.58
Monoclinic, 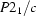

*a* = 9.0559 (10) Å
*b* = 19.382 (2) Å
*c* = 16.5396 (19) Å
β = 92.577 (2)°
*V* = 2900.1 (6) Å^3^
*Z* = 4
Mo *K*α radiation
μ = 0.22 mm^−1^
*T* = 294 K
0.25 × 0.20 × 0.17 mm

#### Data collection

Bruker SMART 1000 CCD diffractometer
Absorption correction: multi-scan (*SADABS*; Bruker, 1998 ▶) *T*_min_ = 0.936, *T*_max_ = 0.974
29195 measured reflections
5245 independent reflections
3748 reflections with *I* > 2σ(*I*)
*R*_int_ = 0.043

#### Refinement

*R*[*F* ^2^ > 2σ(*F* ^2^)] = 0.048
*wR*(*F* ^2^) = 0.139
*S* = 1.06
5245 reflections
379 parameters
H-atom parameters constrained
Δρ_max_ = 0.56 e Å^−3^
Δρ_min_ = −0.29 e Å^−3^

### 

Data collection: *SMART* (Bruker, 1998 ▶); cell refinement: *SAINT-Plus* (Bruker, 1998 ▶); data reduction: *SAINT-Plus*; program(s) used to solve structure: *SIR97* (Altomare *et al.*, 1999 ▶); program(s) used to refine structure: *SHELXL97* (Sheldrick, 2008 ▶); molecular graphics: *ORTEP-3 for Windows* (Farrugia, 1997 ▶) and *SCHAKAL97* (Keller, 1997 ▶); software used to prepare material for publication: *SHELXL97* and *PARST95* (Nardelli, 1995 ▶).

## Supplementary Material

Crystal structure: contains datablocks global, I. DOI: 10.1107/S1600536810038286/lx2176sup1.cif

Structure factors: contains datablocks I. DOI: 10.1107/S1600536810038286/lx2176Isup2.hkl

Additional supplementary materials:  crystallographic information; 3D view; checkCIF report

## Figures and Tables

**Table 1:** Hydrogen-bond geometry (Å, °) *Cg*1, *Cg*2 and *Cg*3 are the centroids of the C1–C6, C21–C26 and C15–C20 phenyl rings, respectively.

*D*—H⋯*A*	*D*—H	H⋯*A*	*D*⋯*A*	*D*—H⋯*A*
C1—H1*B*⋯O4^i^	0.97	2.22	3.191 (4)	178
C26—H26⋯O3^ii^	0.93	2.45	3.373 (3)	174
C32—H32⋯O3^ii^	0.93	2.46	3.369 (4)	168
C1—H1*A*⋯*Cg*1^iii^	0.97	2.84	3.780 (3)	164
C10—H10⋯*Cg*2^iv^	0.93	3.02	3.767 (4)	138
C23—H23⋯*Cg*3^v^	0.93	2.91	3.788 (4)	159

Symmetry codes: (i) 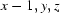; (ii) 
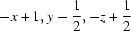
; (iii) 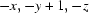; (iv) 
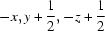
; (v) 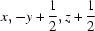.

**Fig. 1.**
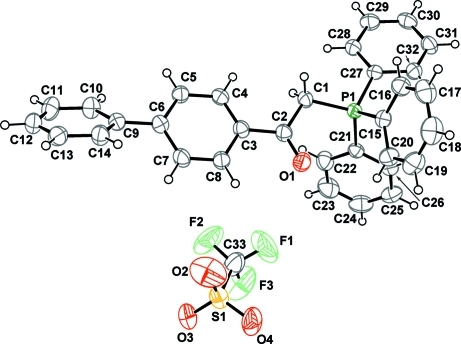
The molecular structure of the title compound showing the atom-labeling scheme. Displacement ellipsoids are drawn at the 40% probability level. H atoms are presented as a small spheres of arbitrary radius.

**Fig. 2.**
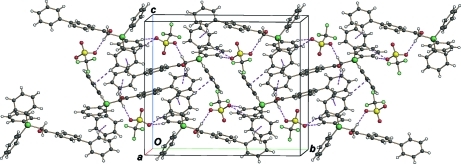
A view of the C—H···O and C—H···π interactions (dashed lines) in the crystal structure of the title compound.

**Fig. 3.**
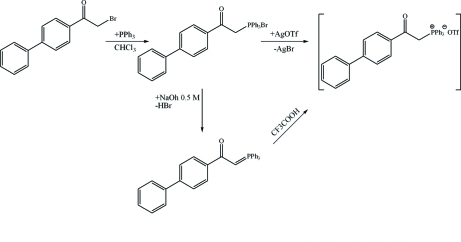
The procedure adopted for the synthesis of the title compound.

## supplementary crystallographic information

### Comment

The synthesis of α–ketostabilized phosphorus ylides has attracted much interest over the last decades due to their stability and ability to act as bifunctional ligands (C- *versus* O-coordination; Kalyanasundari *et al.*, 1995; Kalyanasundari *et al.*, 1999; Laavanya *et al.*, 2001; Vicente *et al.*, 1985). In this respect, the preferred coordination modes of 4-flourobenzyloxymethylenetriphenylphosphorane ylide (FBPPY), 4-chlorobenzyl-oxymethylenetriphenylphosphorane ylide (CBPPY), and 4-methoxybenzoylmethylenetriphenylphosphorane ylide (MOBPPY) to transition metals such as mercury (II), silver (I) and palladium (II) have been recently investigated by our group (Karami, 2007, 2008; Akkurt *et al.*, 2008). As a part of our ongoing study on the synthesis and characterization of new trifluoromethanesulfonate phosphonium ylides, the title compound has been prepared according to the sequence shown in Figure 3 (Burmeister *et al.*, 1973), and its crystal structure is reported herein.

In the title compound (Fig. 1), bond lengths and angles within the phosphonium cation are not unusual and comparable to those observed for the related 4-methoxybenzoylmethyl derivative (Karami & Büyükgüngör, 2009). The P1–C1 bond length (1.798 (2) Å) is significantly longer than that reported in the free ylide (1.711 Å) of formula Ph_3_PC(H)COPh (Shao *et al.*, 1982). The dihedral angle between the benzene rings of the biphenyl group is 42.37 (8)°. Unlike the 4-methoxybenzoylmethyl derivative, the conformation of the cation is not stabilized by intramolecular hydrogen bonds. In the crystal packing (Fig. 2), cations and anions are linked by intermolecular C—H···O hydrogen bonds into chains running parallel to the *b* axis (see Fig. 2 & Table 1). The chains further interact through C—H···π stacking interactions to form layers parallel to the *bc* plane (see  Fig. 2 & Table 1).

### Experimental

The title compound was obtained by reaction of (4–phenylbenzoylmethyl)triphenylphosphonium bromide and AgOTf (OTf :  trifluoromethanesulfonate) in dry acetone in a 1:1 molar ratio under stirring for 12 h (Fig. 3). The precipitate obtained was washed several times with dry diethyl ether and dried in a vacuum. Orange crystals of the title compound suitable for X–ray analysis formed by addition of dry diethyl ether to a chloroform solution. The crystals are air stable and resistant against moisture.

### Refinement

All H atoms were placed in calculated positions and treated as riding on their parent atoms, with C—H = 0.93–0.97 Å, and with *U*_iso_(H) = 1.2 *U*_eq_(C).

### Crystal data

**Table:**

C_32_H_26_OP^+^·CF_3_O_3_S^−^	*F*(000) = 1256
*M**_r_* = 606.58	*D*_x_ = 1.389 Mg m^−^^3^
Monoclinic, *P*2_1_/*c*	Mo *K*α radiation, λ = 0.71073 Å
Hall symbol: -P 2ybc	Cell parameters from 744 reflections
*a* = 9.0559 (10) Å	θ = 6.3–22.4°
*b* = 19.382 (2) Å	µ = 0.22 mm^−^^1^
*c* = 16.5396 (19) Å	*T* = 294 K
β = 92.577 (2)°	Block, orange
*V* = 2900.1 (6) Å^3^	0.25 × 0.20 × 0.17 mm
*Z* = 4	

### Data collection

**Table:**

Bruker SMART 1000 CCD diffractometer	5245 independent reflections
Radiation source: fine-focus sealed tube	3748 reflections with *I* > 2σ(*I*)
graphite	*R*_int_ = 0.043
ω scans	θ_max_ = 25.3°, θ_min_ = 1.6°
Absorption correction: multi-scan (*SADABS*; Bruker, 1998)	*h* = −10→10
*T*_min_ = 0.936, *T*_max_ = 0.974	*k* = −23→23
29195 measured reflections	*l* = −19→19

### Refinement

**Table:**

Refinement on *F*^2^	Primary atom site location: structure-invariant direct methods
Least-squares matrix: full	Secondary atom site location: difference Fourier map
*R*[*F*^2^ > 2σ(*F*^2^)] = 0.048	Hydrogen site location: difference Fourier map
*wR*(*F*^2^) = 0.139	H-atom parameters constrained
*S* = 1.06	*w* = 1/[σ^2^(*F*_o_^2^) + (0.082*P*)^2^] where *P* = (*F*_o_^2^ + 2*F*_c_^2^)/3
5245 reflections	(Δ/σ)_max_ < 0.001
379 parameters	Δρ_max_ = 0.56 e Å^−^^3^
0 restraints	Δρ_min_ = −0.29 e Å^−^^3^

### Special details

**Table:**

Refinement. Refinement of *F*^2^ against ALL reflections. The weighted *R*-factor *wR* and goodness of fit *S* are based on *F*^2^, conventional *R*-factors *R* are based on *F*, with *F* set to zero for negative *F*^2^. The threshold expression of *F*^2^ > σ(*F*^2^) is used only for calculating *R*-factors(gt) *etc*. and is not relevant to the choice of reflections for refinement. *R*-factors based on *F*^2^ are statistically about twice as large as those based on *F*, and *R*- factors based on ALL data will be even larger.

### Fractional atomic coordinates and isotropic or equivalent isotropic displacement parameters (Å^2^)

**Table:**

	*x*	*y*	*z*	*U*_iso_*/*U*_eq_	
P1	0.05841 (6)	0.19921 (3)	0.17248 (4)	0.03733 (18)	
S1	0.78232 (9)	0.42703 (4)	0.27665 (5)	0.0666 (2)	
F1	0.5564 (3)	0.35241 (16)	0.3102 (3)	0.1880 (17)	
F2	0.5301 (3)	0.45799 (15)	0.3327 (2)	0.1577 (13)	
F3	0.6656 (4)	0.39779 (16)	0.41126 (19)	0.1544 (13)	
O1	0.29798 (19)	0.28836 (9)	0.13868 (13)	0.0625 (5)	
O2	0.7193 (4)	0.4436 (2)	0.19884 (16)	0.1420 (13)	
O3	0.8484 (3)	0.48364 (11)	0.31695 (15)	0.0894 (7)	
O4	0.8683 (3)	0.36544 (12)	0.28273 (18)	0.1039 (9)	
C1	0.0380 (3)	0.28830 (11)	0.14394 (15)	0.0435 (6)	
H1A	−0.0207	0.2913	0.0935	0.052*	
H1B	−0.0145	0.3126	0.1851	0.052*	
C2	0.1864 (3)	0.32291 (12)	0.13352 (15)	0.0449 (6)	
C3	0.1899 (2)	0.39764 (12)	0.11621 (14)	0.0433 (6)	
C4	0.0651 (3)	0.43896 (12)	0.11089 (15)	0.0459 (6)	
H4	−0.0267	0.4202	0.1211	0.055*	
C5	0.0757 (3)	0.50804 (12)	0.09048 (15)	0.0453 (6)	
H5	−0.0088	0.5354	0.0883	0.054*	
C6	0.2109 (3)	0.53701 (12)	0.07326 (14)	0.0433 (6)	
C7	0.3360 (3)	0.49527 (13)	0.08168 (17)	0.0535 (7)	
H7	0.4282	0.5140	0.0724	0.064*	
C8	0.3263 (3)	0.42769 (13)	0.10323 (17)	0.0523 (7)	
H8	0.4119	0.4013	0.1093	0.063*	
C9	0.2251 (3)	0.60970 (12)	0.04788 (14)	0.0437 (6)	
C10	0.1499 (3)	0.66280 (13)	0.08446 (16)	0.0549 (7)	
H10	0.0825	0.6526	0.1235	0.066*	
C11	0.1740 (3)	0.73053 (14)	0.06349 (18)	0.0651 (8)	
H11	0.1235	0.7657	0.0888	0.078*	
C12	0.2723 (3)	0.74644 (14)	0.00535 (18)	0.0613 (7)	
H12	0.2913	0.7923	−0.0070	0.074*	
C13	0.3418 (3)	0.69483 (15)	−0.03417 (18)	0.0601 (7)	
H13	0.4048	0.7055	−0.0753	0.072*	
C14	0.3188 (3)	0.62676 (13)	−0.01322 (17)	0.0539 (7)	
H14	0.3667	0.5919	−0.0404	0.065*	
C15	0.1376 (3)	0.14886 (12)	0.09454 (14)	0.0414 (5)	
C16	0.0457 (3)	0.12156 (13)	0.03274 (15)	0.0530 (6)	
H16	−0.0555	0.1299	0.0320	0.064*	
C17	0.1047 (4)	0.08214 (16)	−0.02744 (18)	0.0690 (8)	
H17	0.0437	0.0642	−0.0690	0.083*	
C18	0.2538 (4)	0.06968 (16)	−0.02533 (19)	0.0763 (9)	
H18	0.2933	0.0427	−0.0655	0.092*	
C19	0.3454 (3)	0.09620 (16)	0.03476 (19)	0.0677 (8)	
H19	0.4463	0.0872	0.0353	0.081*	
C20	0.2880 (3)	0.13635 (13)	0.09469 (15)	0.0511 (6)	
H20	0.3505	0.1550	0.1352	0.061*	
C21	0.1644 (2)	0.19295 (12)	0.26621 (14)	0.0398 (5)	
C22	0.1840 (3)	0.24969 (14)	0.31672 (16)	0.0594 (7)	
H22	0.1494	0.2929	0.3003	0.071*	
C23	0.2550 (4)	0.24155 (19)	0.39123 (19)	0.0771 (9)	
H23	0.2690	0.2796	0.4249	0.093*	
C24	0.3053 (3)	0.1785 (2)	0.41654 (18)	0.0728 (9)	
H24	0.3536	0.1739	0.4670	0.087*	
C25	0.2845 (3)	0.12184 (17)	0.36745 (17)	0.0627 (7)	
H25	0.3177	0.0788	0.3851	0.075*	
C26	0.2145 (3)	0.12866 (13)	0.29199 (15)	0.0476 (6)	
H26	0.2011	0.0903	0.2586	0.057*	
C27	−0.1209 (2)	0.16470 (12)	0.19059 (13)	0.0389 (5)	
C28	−0.2382 (3)	0.20647 (13)	0.20946 (15)	0.0473 (6)	
H28	−0.2285	0.2542	0.2078	0.057*	
C29	−0.3701 (3)	0.17695 (15)	0.23093 (18)	0.0591 (7)	
H29	−0.4494	0.2050	0.2431	0.071*	
C30	−0.3841 (3)	0.10673 (15)	0.23429 (17)	0.0576 (7)	
H30	−0.4729	0.0872	0.2488	0.069*	
C31	−0.2677 (3)	0.06509 (14)	0.21634 (16)	0.0555 (7)	
H31	−0.2781	0.0174	0.2188	0.067*	
C32	−0.1356 (3)	0.09315 (12)	0.19477 (15)	0.0469 (6)	
H32	−0.0567	0.0647	0.1831	0.056*	
C33	0.6237 (4)	0.4073 (2)	0.3325 (3)	0.0963 (13)	

### Atomic displacement parameters (Å^2^)

**Table:**

	*U*^11^	*U*^22^	*U*^33^	*U*^12^	*U*^13^	*U*^23^
P1	0.0344 (3)	0.0359 (3)	0.0418 (3)	−0.0018 (2)	0.0030 (2)	−0.0002 (3)
S1	0.0717 (5)	0.0581 (5)	0.0714 (5)	−0.0069 (4)	0.0203 (4)	−0.0080 (4)
F1	0.116 (2)	0.126 (2)	0.327 (5)	−0.0639 (19)	0.070 (3)	−0.014 (3)
F2	0.0997 (18)	0.150 (2)	0.229 (3)	0.0702 (17)	0.0710 (19)	0.072 (2)
F3	0.179 (3)	0.161 (3)	0.130 (2)	0.054 (2)	0.077 (2)	0.072 (2)
O1	0.0444 (10)	0.0477 (11)	0.0966 (15)	0.0018 (8)	0.0148 (10)	0.0106 (10)
O2	0.148 (3)	0.214 (4)	0.0626 (17)	−0.034 (2)	−0.0075 (17)	0.0103 (19)
O3	0.1034 (17)	0.0534 (12)	0.1110 (19)	−0.0142 (12)	0.0002 (14)	−0.0081 (12)
O4	0.1033 (18)	0.0602 (14)	0.153 (3)	0.0123 (13)	0.0578 (17)	−0.0148 (14)
C1	0.0414 (13)	0.0375 (13)	0.0520 (14)	−0.0040 (10)	0.0054 (11)	0.0025 (10)
C2	0.0416 (14)	0.0432 (13)	0.0507 (14)	−0.0023 (11)	0.0098 (11)	0.0003 (11)
C3	0.0420 (13)	0.0422 (13)	0.0462 (14)	−0.0041 (10)	0.0084 (10)	−0.0010 (10)
C4	0.0384 (13)	0.0478 (14)	0.0517 (14)	−0.0064 (11)	0.0058 (11)	0.0020 (11)
C5	0.0424 (13)	0.0436 (14)	0.0503 (14)	0.0012 (11)	0.0061 (11)	0.0007 (11)
C6	0.0464 (14)	0.0426 (13)	0.0414 (13)	−0.0025 (11)	0.0066 (10)	−0.0041 (10)
C7	0.0417 (14)	0.0457 (15)	0.0742 (18)	−0.0067 (11)	0.0147 (13)	0.0010 (13)
C8	0.0387 (13)	0.0423 (14)	0.0769 (19)	0.0013 (11)	0.0137 (12)	−0.0007 (12)
C9	0.0452 (13)	0.0424 (13)	0.0435 (13)	−0.0021 (11)	0.0018 (11)	−0.0002 (10)
C10	0.0666 (17)	0.0495 (16)	0.0497 (15)	0.0016 (13)	0.0129 (13)	−0.0033 (12)
C11	0.091 (2)	0.0433 (15)	0.0612 (18)	0.0113 (15)	0.0022 (16)	−0.0048 (13)
C12	0.0691 (18)	0.0428 (15)	0.0709 (19)	−0.0043 (13)	−0.0090 (15)	0.0096 (13)
C13	0.0476 (15)	0.0601 (18)	0.0730 (19)	−0.0016 (13)	0.0078 (13)	0.0194 (15)
C14	0.0504 (15)	0.0471 (15)	0.0652 (17)	0.0008 (12)	0.0148 (13)	0.0026 (12)
C15	0.0467 (14)	0.0397 (13)	0.0382 (13)	0.0008 (10)	0.0056 (10)	0.0023 (10)
C16	0.0560 (15)	0.0547 (16)	0.0481 (15)	−0.0007 (12)	0.0000 (12)	−0.0033 (12)
C17	0.088 (2)	0.068 (2)	0.0516 (17)	−0.0058 (17)	0.0021 (15)	−0.0153 (14)
C18	0.102 (3)	0.071 (2)	0.0576 (19)	0.0077 (19)	0.0256 (18)	−0.0164 (15)
C19	0.0650 (18)	0.0716 (19)	0.068 (2)	0.0138 (15)	0.0215 (15)	−0.0001 (16)
C20	0.0474 (15)	0.0577 (16)	0.0488 (15)	0.0050 (12)	0.0083 (12)	−0.0014 (12)
C21	0.0345 (12)	0.0438 (13)	0.0413 (13)	−0.0028 (10)	0.0044 (9)	−0.0023 (10)
C22	0.0720 (19)	0.0517 (16)	0.0539 (17)	−0.0014 (14)	−0.0032 (14)	−0.0094 (13)
C23	0.087 (2)	0.085 (2)	0.0588 (19)	−0.0074 (19)	−0.0087 (16)	−0.0219 (17)
C24	0.0621 (19)	0.107 (3)	0.0481 (17)	0.0019 (18)	−0.0105 (14)	−0.0032 (17)
C25	0.0531 (16)	0.078 (2)	0.0571 (18)	0.0112 (14)	−0.0008 (13)	0.0138 (15)
C26	0.0446 (14)	0.0495 (15)	0.0485 (14)	0.0015 (11)	0.0005 (11)	0.0010 (11)
C27	0.0351 (12)	0.0427 (13)	0.0387 (12)	−0.0022 (10)	−0.0001 (10)	0.0016 (10)
C28	0.0417 (13)	0.0434 (14)	0.0567 (15)	−0.0007 (11)	0.0018 (11)	−0.0025 (11)
C29	0.0372 (14)	0.0685 (19)	0.0719 (19)	0.0000 (13)	0.0076 (13)	−0.0009 (14)
C30	0.0399 (14)	0.0691 (19)	0.0642 (18)	−0.0146 (13)	0.0041 (12)	0.0028 (14)
C31	0.0545 (16)	0.0468 (15)	0.0649 (18)	−0.0150 (12)	0.0011 (13)	0.0038 (12)
C32	0.0434 (13)	0.0422 (14)	0.0554 (15)	−0.0022 (11)	0.0039 (11)	0.0027 (11)
C33	0.078 (2)	0.077 (3)	0.137 (4)	0.019 (2)	0.036 (2)	0.028 (2)

### Geometric parameters (Å, °)

**Table:**

P1—C21	1.790 (2)	C13—H13	0.9300
P1—C15	1.792 (2)	C14—H14	0.9300
P1—C27	1.794 (2)	C15—C20	1.383 (3)
P1—C1	1.798 (2)	C15—C16	1.393 (3)
S1—O3	1.404 (2)	C16—C17	1.381 (4)
S1—O2	1.421 (3)	C16—H16	0.9300
S1—O4	1.427 (2)	C17—C18	1.371 (4)
S1—C33	1.783 (4)	C17—H17	0.9300
F1—C33	1.273 (5)	C18—C19	1.366 (4)
F2—C33	1.298 (4)	C18—H18	0.9300
F3—C33	1.354 (5)	C19—C20	1.380 (4)
O1—C2	1.212 (3)	C19—H19	0.9300
C1—C2	1.519 (3)	C20—H20	0.9300
C1—H1A	0.9700	C21—C26	1.387 (3)
C1—H1B	0.9700	C21—C22	1.388 (3)
C2—C3	1.477 (3)	C22—C23	1.373 (4)
C3—C4	1.385 (3)	C22—H22	0.9300
C3—C8	1.391 (3)	C23—C24	1.363 (5)
C4—C5	1.385 (3)	C23—H23	0.9300
C4—H4	0.9300	C24—C25	1.374 (4)
C5—C6	1.387 (3)	C24—H24	0.9300
C5—H5	0.9300	C25—C26	1.380 (4)
C6—C7	1.394 (3)	C25—H25	0.9300
C6—C9	1.477 (3)	C26—H26	0.9300
C7—C8	1.361 (3)	C27—C28	1.382 (3)
C7—H7	0.9300	C27—C32	1.395 (3)
C8—H8	0.9300	C28—C29	1.385 (3)
C9—C10	1.388 (3)	C28—H28	0.9300
C9—C14	1.389 (3)	C29—C30	1.368 (4)
C10—C11	1.378 (4)	C29—H29	0.9300
C10—H10	0.9300	C30—C31	1.371 (4)
C11—C12	1.374 (4)	C30—H30	0.9300
C11—H11	0.9300	C31—C32	1.376 (3)
C12—C13	1.365 (4)	C31—H31	0.9300
C12—H12	0.9300	C32—H32	0.9300
C13—C14	1.382 (4)		
			
C21—P1—C15	111.69 (11)	C16—C15—P1	119.27 (19)
C21—P1—C27	106.52 (10)	C17—C16—C15	120.0 (3)
C15—P1—C27	108.17 (11)	C17—C16—H16	120.0
C21—P1—C1	109.71 (11)	C15—C16—H16	120.0
C15—P1—C1	111.96 (11)	C18—C17—C16	119.5 (3)
C27—P1—C1	108.60 (11)	C18—C17—H17	120.3
O3—S1—O2	113.5 (2)	C16—C17—H17	120.3
O3—S1—O4	113.63 (16)	C19—C18—C17	121.2 (3)
O2—S1—O4	116.7 (2)	C19—C18—H18	119.4
O3—S1—C33	104.93 (19)	C17—C18—H18	119.4
O2—S1—C33	102.5 (2)	C18—C19—C20	119.9 (3)
O4—S1—C33	103.55 (16)	C18—C19—H19	120.0
C2—C1—P1	111.90 (16)	C20—C19—H19	120.0
C2—C1—H1A	109.2	C19—C20—C15	120.0 (3)
P1—C1—H1A	109.2	C19—C20—H20	120.0
C2—C1—H1B	109.2	C15—C20—H20	120.0
P1—C1—H1B	109.2	C26—C21—C22	119.7 (2)
H1A—C1—H1B	107.9	C26—C21—P1	118.85 (18)
O1—C2—C3	122.0 (2)	C22—C21—P1	121.1 (2)
O1—C2—C1	119.1 (2)	C23—C22—C21	119.4 (3)
C3—C2—C1	118.8 (2)	C23—C22—H22	120.3
C4—C3—C8	118.4 (2)	C21—C22—H22	120.3
C4—C3—C2	123.7 (2)	C24—C23—C22	121.0 (3)
C8—C3—C2	117.9 (2)	C24—C23—H23	119.5
C5—C4—C3	120.6 (2)	C22—C23—H23	119.5
C5—C4—H4	119.7	C23—C24—C25	120.0 (3)
C3—C4—H4	119.7	C23—C24—H24	120.0
C4—C5—C6	120.9 (2)	C25—C24—H24	120.0
C4—C5—H5	119.6	C24—C25—C26	120.2 (3)
C6—C5—H5	119.6	C24—C25—H25	119.9
C5—C6—C7	117.7 (2)	C26—C25—H25	119.9
C5—C6—C9	122.2 (2)	C25—C26—C21	119.7 (2)
C7—C6—C9	120.1 (2)	C25—C26—H26	120.2
C8—C7—C6	121.5 (2)	C21—C26—H26	120.2
C8—C7—H7	119.2	C28—C27—C32	119.7 (2)
C6—C7—H7	119.2	C28—C27—P1	121.99 (18)
C7—C8—C3	120.8 (2)	C32—C27—P1	117.89 (17)
C7—C8—H8	119.6	C27—C28—C29	119.7 (2)
C3—C8—H8	119.6	C27—C28—H28	120.1
C10—C9—C14	117.9 (2)	C29—C28—H28	120.1
C10—C9—C6	122.2 (2)	C30—C29—C28	120.3 (2)
C14—C9—C6	119.9 (2)	C30—C29—H29	119.9
C11—C10—C9	120.7 (3)	C28—C29—H29	119.9
C11—C10—H10	119.7	C29—C30—C31	120.2 (2)
C9—C10—H10	119.7	C29—C30—H30	119.9
C12—C11—C10	120.3 (3)	C31—C30—H30	119.9
C12—C11—H11	119.8	C30—C31—C32	120.6 (2)
C10—C11—H11	119.8	C30—C31—H31	119.7
C13—C12—C11	119.9 (3)	C32—C31—H31	119.7
C13—C12—H12	120.1	C31—C32—C27	119.5 (2)
C11—C12—H12	120.1	C31—C32—H32	120.3
C12—C13—C14	120.1 (3)	C27—C32—H32	120.3
C12—C13—H13	119.9	F1—C33—F2	109.2 (4)
C14—C13—H13	119.9	F1—C33—F3	105.9 (4)
C13—C14—C9	120.9 (2)	F2—C33—F3	104.8 (4)
C13—C14—H14	119.5	F1—C33—S1	114.5 (3)
C9—C14—H14	119.5	F2—C33—S1	112.4 (3)
C20—C15—C16	119.4 (2)	F3—C33—S1	109.3 (3)
C20—C15—P1	121.34 (19)		

### Hydrogen-bond geometry (Å, °)

**Table:**

*Cg*1, *Cg*2 and *Cg*3 are the centroids of the C1–C6, C21–C26 and C15–C20 phenyl rings, respectively.

**Table:**

*D*—H···*A*	*D*—H	H···*A*	*D*···*A*	*D*—H···*A*
C1—H1B···O4^i^	0.97	2.22	3.191 (4)	178
C26—H26···O3^ii^	0.93	2.45	3.373 (3)	174
C32—H32···O3^ii^	0.93	2.46	3.369 (4)	168
C1—H1A···Cg1^iii^	0.97	2.84	3.780 (3)	164
C10—H10···Cg2^iv^	0.93	3.02	3.767 (4)	138
C23—H23···Cg3^v^	0.93	2.91	3.788 (4)	159

Symmetry codes: (i) *x*−1, *y*, *z*; (ii) −*x*+1, *y*−1/2, −*z*+1/2; (iii) −*x*, −*y*+1, −*z*; (iv) −*x*, *y*+1/2, −*z*+1/2; (v) *x*, −*y*+1/2, *z*+1/2.
